# Classification of Tomato Fruit Using Yolov5 and Convolutional Neural Network Models

**DOI:** 10.3390/plants12040790

**Published:** 2023-02-09

**Authors:** Quoc-Hung Phan, Van-Tung Nguyen, Chi-Hsiang Lien, The-Phong Duong, Max Ti-Kuang Hou, Ngoc-Bich Le

**Affiliations:** 1 Department of Mechanical Engineering, National United University, Miaoli 360302, Taiwan; 2 Department of Mechanical Engineering, HCMC University of Technology and Education, Ho Chi Minh City 700000, Vietnam; 3 School of Biomedical Engineering, International University, Ho Chi Minh City 700000, Vietnam; 4 Vietnam National University HCMC, Ho Chi Minh City 700000, Vietnam

**Keywords:** deep learning, convolution neural network, Yolov5, tomato fruit classification

## Abstract

Four deep learning frameworks consisting of Yolov5m and Yolov5m combined with ResNet50, ResNet-101, and EfficientNet-B0, respectively, are proposed for classifying tomato fruit on the vine into three categories: ripe, immature, and damaged. For a training dataset consisting of 4500 images and a training process with 200 epochs, a batch size of 128, and an image size of 224 × 224 pixels, the prediction accuracy for ripe and immature tomatoes is found to be 100% when combining Yolo5m with ResNet-101. Meanwhile, the prediction accuracy for damaged tomatoes is 94% when using Yolo5m with the Efficient-B0 model. The ResNet-50, EfficientNet-B0, Yolov5m, and ResNet-101 networks have testing accuracies of 98%, 98%, 97%, and 97%, respectively. Thus, all four frameworks have the potential for tomato fruit classification in automated tomato fruit harvesting applications in agriculture.

## 1. Introduction

Fruit harvesting is labor-intensive and time-consuming work. However, with the development of artificial intelligence (AI), much of this work can now be performed by robots. Robotic harvesting comprises two main steps: fruit detection using a computer vision system and fruit picking using a robot arm. Of the two steps, fruit detection is the most crucial, since it is vital that only the fruit which are ripe and ready for consumption are harvested, while the remainder are left on the branch or vine to mature. Many techniques have been developed for fruit detection over the last decade. Conventional techniques rely mainly on color, texture, shape, and other shallow features of the image for detection [1,2,3,4]. However, the detection accuracy of such methods is heavily dependent on the illumination conditions. Moreover, as the detection algorithms are complicated and have many fixed thresholds, it is difficult to adapt them to other fruits and/or environments. Thus, the development of AI technology has prompted significant interest in the potential for applying machine learning to computer vision tasks, such as harvesting, in agriculture. Many machine learning methods [5,6,7] have been proposed for fruit classification based on color detection, edge detection, etc. Zhao et al. [8] proposed the AdaBoost algorithm combined with the average pixel value (APV) for tomato fruit detection. Based on the shape, texture, color properties, and Haar-like features of ordinary color images, the proposed algorithm has an accuracy of 96.5% when detecting ripe tomatoes. Luo et al. [9] used the AdaBoost framework and multiple color components obtained from the vision sensor to automatically identify clusters of ripe grapes on a farm. The proposed method has an accuracy of 96.56%. Liu et al. [10] employed an automatic tomato detection method for ordinary color images. The histograms of oriented gradients (HOG) method was used to train the support vector machine (SVM) classifier. A coarse-to-fine scanner was used for tomato detection, false color removal (FCR) to remove false-positive rejection, and non-maximum suppression (NMS) to merge overlapping results. The F1, recall, and precision scores were 92.15%, 94.41%, and 90.00%, respectively. However, most methods are complicated in design, have low-level abstraction, and are adapted only to certain specific conditions. Furthermore, machine learning methods have poor robustness in complex scenes, and it is extremely difficult to transfer these methods from one kind of fruit to another.

The emergence of deep learning technology has led to the development of many machine learning models for image recognition purposes. Among these models, convolutional neural networks (CNN) are one of the most commonly applied. Many CNN architectures have been developed, including AlexNet [11], GoogLeNet [12], VGGNet [13], and ResNet [14]. Zhang et al. [15] proposed several frameworks based on AlexNet, ResNet, and GoogLeNet architectures, respectively, to identify tomato leaf disease. Aversano et al. [16] similarly employed VGG architecture to perform tomato disease classification. Karthik et al. [17] proposed a new residual CNN for disease detection in tomato leaves.

Image detection systems based on CNNs fall into two general categories. In the first category, a series of candidate frames are generated as samples, and the samples are then classified by CNN models such as R-CNN (Regions with CNN features) [18], Faster R-CNN [19], and R-FCN (Regions-based Fully Convolutional Network) [20]. Methods in the second category transfer the problem of object bounding box location into a regression problem, such that candidate boxes do not need to be generated. Such methods include SSD (Single-Shot multi-box Detector) [21] and YOLO (You Only Look Once) [22]. Hu et al. [23] used Faster R-CNN to detect candidate ripe tomato regions in complex greenhouse environments. Fuentes et al. [24] compared three common detector architectures, namely Faster R-CNN, R-FCN, and SSD, for tomato plant disease detection. Mirhaji et al. [25] used the YOLO-v4 detection model to detect and count ripe Dezful native oranges in an orchard in southwestern Iran. The precision, recall, F1-score, and mAP were shown to be 91.23%, 92.8%, 92%, and 90.8%, respectively. Chen et al. [26] developed a modified Yolov3 for diseased cherry tomato detection. Based on an improved dual-path network and K-means++, the model obtained a precision rate of 94.29%, a recall rate of 94.07%, and an F1 score of 94.18%. Overall, the results presented in [23,24,25,26] confirm the feasibility of utilizing CNN for fruit and vegetable detection. However, the detection accuracy of the proposed methods is still relatively poor. Hence, more sophisticated CNN techniques for crop detection are required.

Tomatoes are rich in vitamins and form the basis of many different dishes around the world. With thin skins, succulent flesh, high sweetness, and a high nutritional value, they are one of the most widely cultivated fruits globally [27]. In Taiwan, almost 50 different types of tomatoes are planted throughout the year, with the most common varieties being black tomatoes, Momotaro tomatoes, golden tomatoes, and cherry tomatoes. Tomatoes are cultivated extensively throughout Taiwan. About 5000 hectares of tomato is cultivated in Taiwan, with the main growing areas located in Chiayi, Kaohsiung, Tainan, Yunlin, and Nantou. Moreover, the annual tomato output value is around TWD 30 billion and is increasing year by year [28]. Consequently, many tomato detection methods based on CNN models have been proposed. Wang et al. [29] used Faster R-CNN with ResNet50 architecture to improve the detection accuracy for young tomato fruit. The results showed that the MAP of the proposed method reached as high as 98.46%. Zu et al. [30] applied a masked R-CNN model with automatic image acquisition to the detection and segmentation of mature green tomatoes. It was shown that the F1 scores of the bounding box and mask region were both 92.0%. Lewal [31] used a modified Yolov3 model referred to as the YOLO-tomato model for detecting tomatoes in complex environmental conditions. The maximum detection accuracy was shown to be 99.5%. Liu et al. [32] performed tomato detection using the YOLO-tomato model with a new circular bounding box (C-Bbox) method. The maximum detection accuracy was shown to be 94.58%.

The techniques described in [29,30,31,32] provide useful and reliable solutions for tomato detection. However, more work is required to improve their detection performance in complex real-world greenhouse environments. In a previous study, the present group proposed a CNN-based technique for strawberry disease classification with an accuracy of 98–100% [33]. Building on the results obtained in [33], the present study combines the Yolov5 medium with four different CNN classification models (Yolo5m, ResNet50, ResNet-101 and EfficientNet-B0) to classify three states of tomatoes on the vine into three categories: ripe, immature, and damaged.

## 2. Results and Discussion

**Figure 1:**
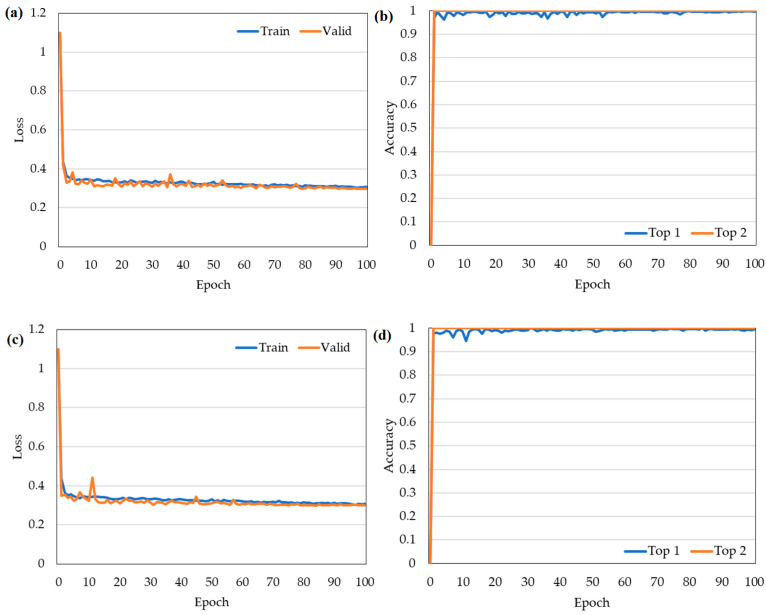

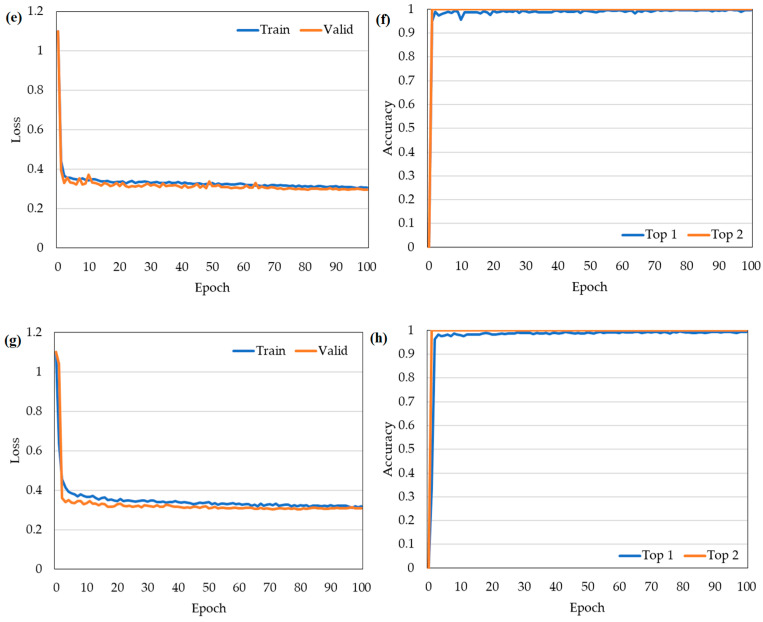
Training loss (**left**) and accuracy (**right**) of the four models: (**a**,**b**) Yolov5m; (**c**,**d**) ResNet-50; (**e**,**f**) ResNet-101; and (**g**,**h**) EfficientNet-B0.

Figure 1 shows the training loss and accuracy of the four classification models (Yolov5m, ResNet-50, ResNet-101, and Efficient Net-B0). The structural parameters and Top 1 and Top 2 accuracies of the four models are listed in Table 1. As shown, the Top 1 accuracy of the four models lies in the range of 93.3–99.7% after 100 training epochs. Of the four models, Yolov5m (Figure 1b) and ResNet-101 (Figure 1d) achieve the highest Top 1 accuracies of 99.7%, while EfficientNet-B0 (Figure 1f) and ResNet-50 (Figure 1h) have the lowest Top 1 accuracies of 99.3%. All four models have a Top 2 accuracy of 100%. EfficientNet-B0 has the fastest training time of 50 min, while ResNet-101 has the slowest training time of 1 h 13 min (Table 1). Overall, the Yolov5m model provides the best tradeoff between the accuracy (0.997) and the training time (52 min). Notably, none of the four models show signs of overfitting or underfitting. Hence, the effectiveness of the data augmentation process is confirmed.

**Figure 2:**
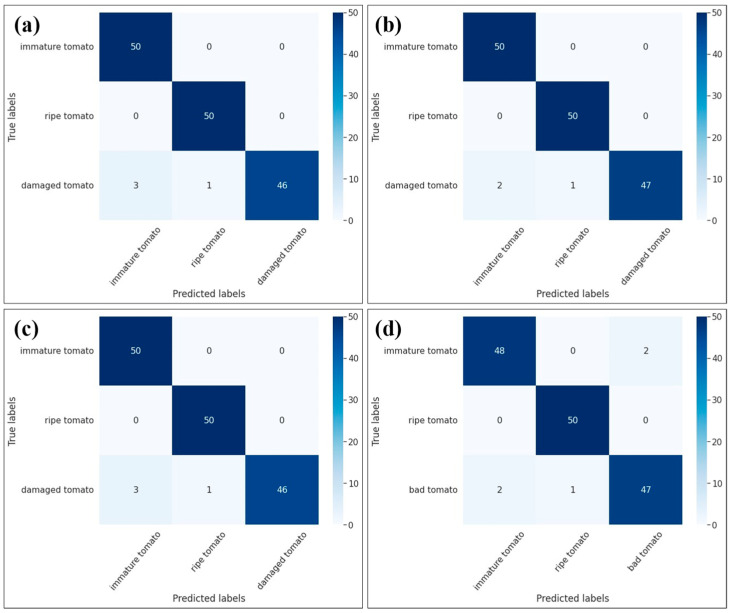
Confusion matrices for (**a**) standalone Yolov5m model, (**b**) Yolov5 with ResNet-50, (**c**) Yolov5 with ResNet-101, and (**d**) Yolov5 with Efficient-B0.

Figure 2 shows the confusion matrices of the training results for the four models. As shown in Figure 2a, the Yolov5m model achieved a classification accuracy rate of 100% for ripe tomatoes, 100% for immature tomatoes, and 92% for damaged tomatoes (including a 6% error for immature tomatoes and 2% error for ripe tomatoes). The relatively poor accuracy of the classification model for the damaged tomatoes can be explained by the fact that the color characteristics of the damaged tomatoes are similar to those of the ripe and immature tomatoes. For the Yolo5m with ResNet50 model (Figure 2b), the classification accuracy was 100% for both ripe and immature tomatoes and 94% for damaged tomatoes (see Figure 2b). In other words, the classification accuracy for damaged tomatoes was improved by 2% compared to that of the Yolo5m model alone. As shown in Figure 2c, the Yolo5m with ResNet-101 model achieved a classification accuracy of 100% for immature and ripe tomatoes and 92% for damaged tomatoes. In other words, the accuracy of the combined model was the same as that of the standalone Yolov5m model. Thus, combining Yolo5m with ResNet-101 not only failed to improve the accuracy but also increased the training time. Finally, the Yolo5m with Efficient-B0 model achieved a classification accuracy of 100% for ripe tomatoes, 96% for immature tomatoes, and 94% for damaged tomatoes (Figure 2d). Thus, the classification performance is generally inferior to that of the standalone Yolov5m model. However, its training time is the shortest of the four models, since it contains just 4.0 M parameters (Table 1).

**Figure 3:**
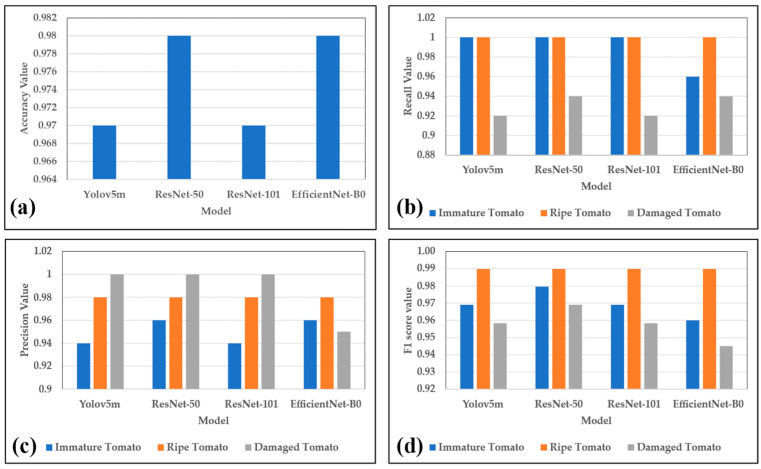
Accuracy, recall, precision, and F1 scores of the four models. (**a**) Accuracy, (**b**) recall, (**c**) precision, and (**d**) F1 score.

Figure 3 shows the accuracy, recall, precision, and F1 score metrics of the four models in the testing stage. As shown in Figure 3a, ResNet-50 and EfficientNet-B0 have the highest accuracies of 98%, while Yolov5m and ResNet-101 have the lowest accuracies of 97%. All four models have a recall of 100% for ripe tomatoes, as shown in Figure 3b. Yolov5m, ResNet-50, and ResNet-101 also have recall values of 1 for immature tomatoes. However, the recall value of EfficientNet-B0 falls to 0.96. Meanwhile, the recall values for damaged tomatoes vary in the range of 0.92 to 0.94 across the four models. As shown in Figure 3c, all four models have a precision of 0.98 for ripe tomatoes. Yolov5m, ResNet-50, and ResNet-101 have a precision of 1 for damaged tomatoes. However, EfficientNet-B0 has a lower precision of 0.95. The precision values of the four models for immature tomatoes vary from 0.94 to 0.96. All four models have an F1 score of 0.99 for ripe tomatoes (see Figure 3d). In other words, the models tend to predict the ripe tomato state more accurately than the other states. The F1 scores for the immature tomatoes and damaged tomatoes vary in the ranges of 0.96–0.98 and 0.93–0.97, respectively.

**Figure 4:**
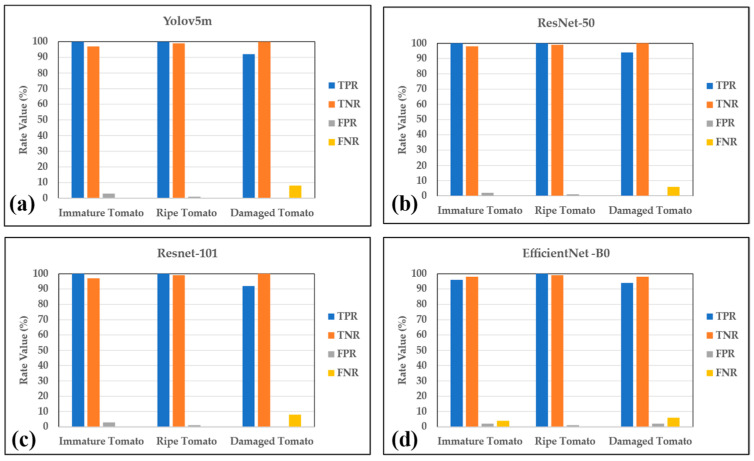
TPR, TNR, FPR, and FNR values of (**a**) Yolov5m, (**b**) Resnet-50, (**c**) Resnet-101, and (**d**) EfficientNet-B0 models.

Figure 4 shows the TPR, TNR, FPR, and FNR values of the four models in the testing stage. As shown, the TPR values of immature and ripe tomatoes are high, with a range of 96–100%. However, damaged tomatoes have lower TPR values of 92–94%. Similar to the TPR value, the TNR of the three categories also has a high value of 98–99%. The FPR values of Yolov5m, ResNet-50, ResNet-101, and EfficientNet-B0 models are low values of 0–3%, 0–2%, 0–3%, and 1–2%, respectively. Finally, the FNR has a low value of 0–8%. The ResNet-101 model has the lowest value of 0–6%. The four models are not underfitted or overfitted, thus posing less risk of confusion and error.

## 3. Materials and Methods

### 3.1. Tomato State Dataset

Tomato images were collected from tomato farms in Miaoli County, Taiwan, and the Asian Vegetable Research and Development Center (AVRDC) in Tainan, Taiwan, using an iPhone 11. The images were collected from many different angles and distances, at different times of day, and in a variety of weather conditions in order to increase the efficiency and accuracy of the deep learning model. A total of 1508 images were obtained with a size of 3024 × 4032 pixels, a bit depth of 24, and a dpi resolution of 72 in both the horizontal and the vertical directions.

**Figure 5:**
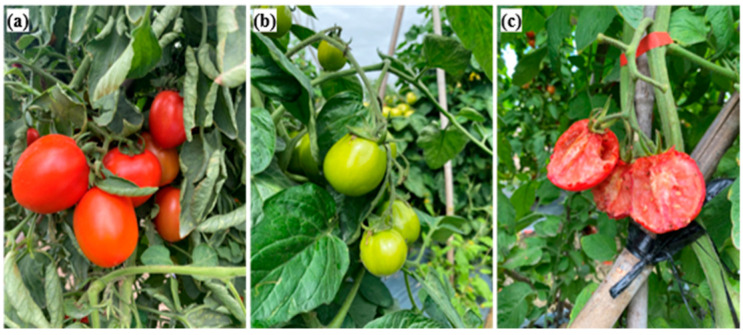
Illustrative images of tomatoes in three states: (**a**) ripe, (**b**) immature, and (**c**) damaged.

**Figure 6:**
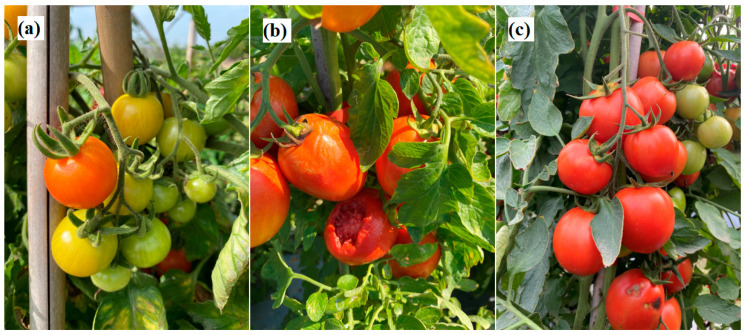
Illustrative images of ripe tomatoes taken at different times of day: (**a**) 9:00 a.m., (**b**) 12:00 p.m., and (**c**) 5:00 p.m.

**Figure 7:**
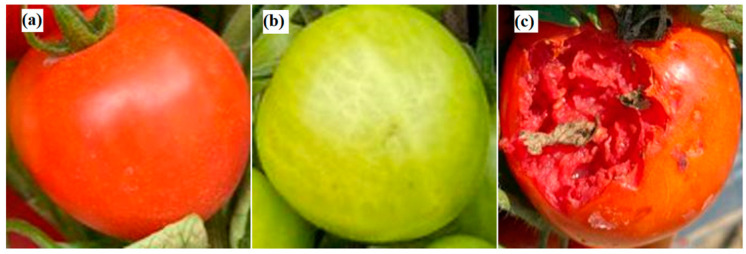
Cropped and normalized images of tomatoes in three states: (**a**) ripe, (**b**) immature, and (**c**) damaged.

Figure 5 presents typical images acquired for three tomato states: ripe, immature, and damaged. As shown, the ripe tomatoes have an orange to red color, the immature tomatoes are green, and the damaged tomatoes have an irregular shape and obvious physical damage. To improve the accuracy of the training model, the tomato images were acquired at three different times of the day (9.00 a.m., 12.00 p.m., and 5.00 p.m.) in order to capture the effects of different illumination conditions. As shown in Figure 6, the images captured at midday showed intense brightness and deep shadows, while the images taken in the afternoon were darker and more uniform in color and intensity. The captured images were cropped and normalized to a size of 224 × 224 pixels to optimize the model training process (see Figure 7).

### 3.2. Data Augmentation

**Figure 8:**
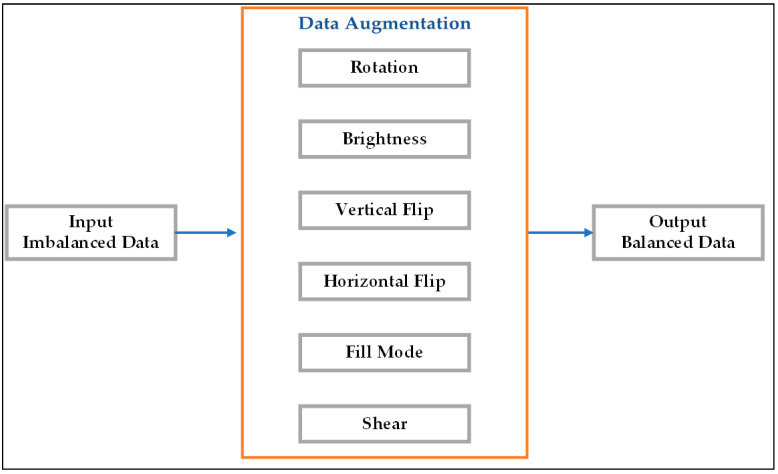
Data augmentation process.

**Figure 9:**
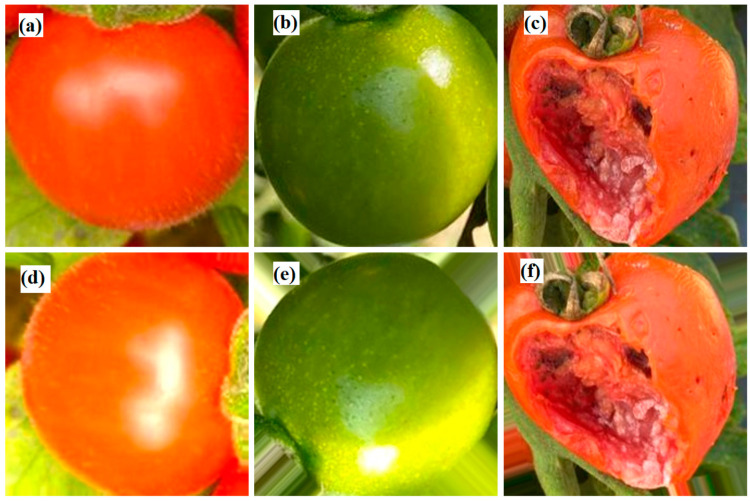
The results after data augmentation. (**a**) Original ripe tomato, (**b**) original immature tomato, (**c**) original damaged tomato, (**d**) data augmentation of ripe tomato, (**e**) data augmentation of immature tomato, and (**f**) data augmentation of damaged tomato.

Following the cropping and normalization process, the tomato image database contained 2176 images of immature tomatoes, 1753 images of ripe tomatoes, and 557 images of damaged tomatoes. For each category, 50 images were used for testing, while the remaining images (2127 images for immature tomatoes, 1703 images for ripe tomatoes, and 507 images for damaged tomatoes) were retained for training and validation purposes. The testing dataset thus contained very different numbers of images for each category. Accordingly, a data argumentation process was performed to balance the dataset and increase the classification accuracy. As shown in Figure 8, the augmentation process included image rotation from 0 to 90°, brightness adjustment from 1.0 to 2.0, vertical and horizontal flipping, filling using the “nearest” mode, and shearing with a range of 0.2. Figure 9 shows the typical augmentation results obtained for the ripe, immature, and damaged tomato categories, respectively. After balancing, a dataset was constructed consisting of 1500 images for each category.

### 3.3. Yolov5 Network Model

**Figure 10:**
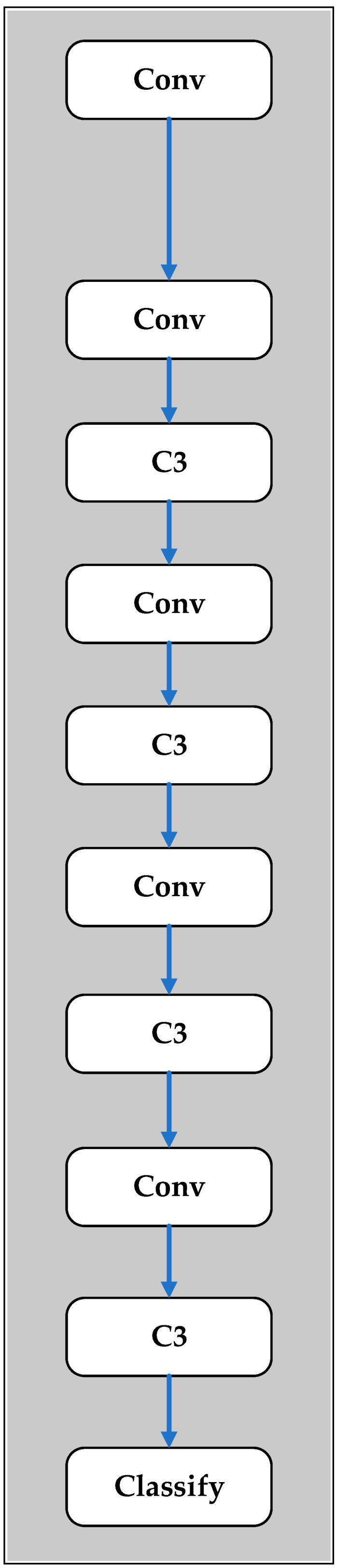
Backbone structure of Yolov5m model.

Yolov5 is an object detection network model that belongs to the Yolo family of models. The first three versions of Yolo were developed by Joseph Redmon between 2015 and 2018, while Yolov4 was released by Alexey Bochkovskiy in 2020 with an improved speed and accuracy [34]. Yolov5 was published by Glenn Jocher in 2020 with initial comparisons showing the same accuracy as Yolov4 but a faster prediction speed [35]. Yolov5 has five network model versions: Yolov5n, Yolov5s, Yolov5m, Yolov5l, and Yolov5x. While Yolov5n has the fastest calculation speed, its average precision is the lowest. Conversely, Yolov5x has the slowest calculation speed but the highest average precision [36]. In the present study, the tomato state classification system was implemented using the Yolov5m model. As shown in Figure 10, the backbone structure consisted of a Conv (Convolutional) layer, a C3 (Cross Stage Partial Networks Bottleneck with 3 convolutions) layer, and a classification layer. In total, the model consisted of 212 layers with 11.7 million parameters and 30.9 GFLOPs (Giga Floating Point Operations Per Second).

### 3.4. Residual Network (ResNet-50 and ResNet-101)

**Figure 11:**
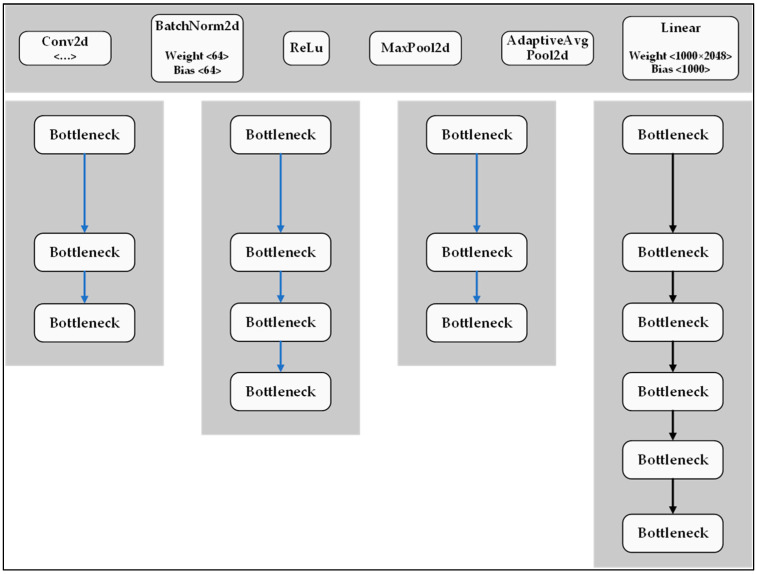
Structure of ResNet-50 model.

Deep CNN networks are affected by several limitations, including a time-consuming optimization process, a vanishing gradient problem, and degradation problems [37]. Residual Network (ResNet) improves many of these limitations and provides the ability to solve complicated tasks with an increased accuracy [38]. Although it is regarded as a deep network when implemented with 152 layers, it only has around 26 million parameters [39]. ResNet has a convolution block that uses the same 3 × 3 filter as InceptionNet. The convolution block consists of 2 convolution branches, where 1 branch applies a 1 × 1 convolution before adding it directly to the other branch. The identity block does not apply the 1 × 1 convolution but directly adds the value of the former branch to the other branch. Figure 11 shows the basic structure of the ResNet-50 model. To minimize the training time, the model is implemented using bottlenecks as the basic building block, where each building block consists of convolutional layers (2 layers of 1 × 1 and 1 layer of 3 × 3 in the middle) and keeps the original features of the images. Moreover, the ResNet-50 model uses a stack of three layers rather than the two layers employed in the ResNet-34 model. The three layers comprise 1 × 1, 3 × 3, and 1 × 1 convolutions, where the 1 × 1 layers are responsible for reducing and then increasing the dimensions, while the 3 × 3 layer serves as a bottleneck [40]. In this study, the ResNet-50 model is run based on the YOLOv5 environment. It is noted that the proposed ResNet-50 model includes a total of 151 layers, 23.5 million parameters, and 67.5 GFLOPs.

**Figure 12:**
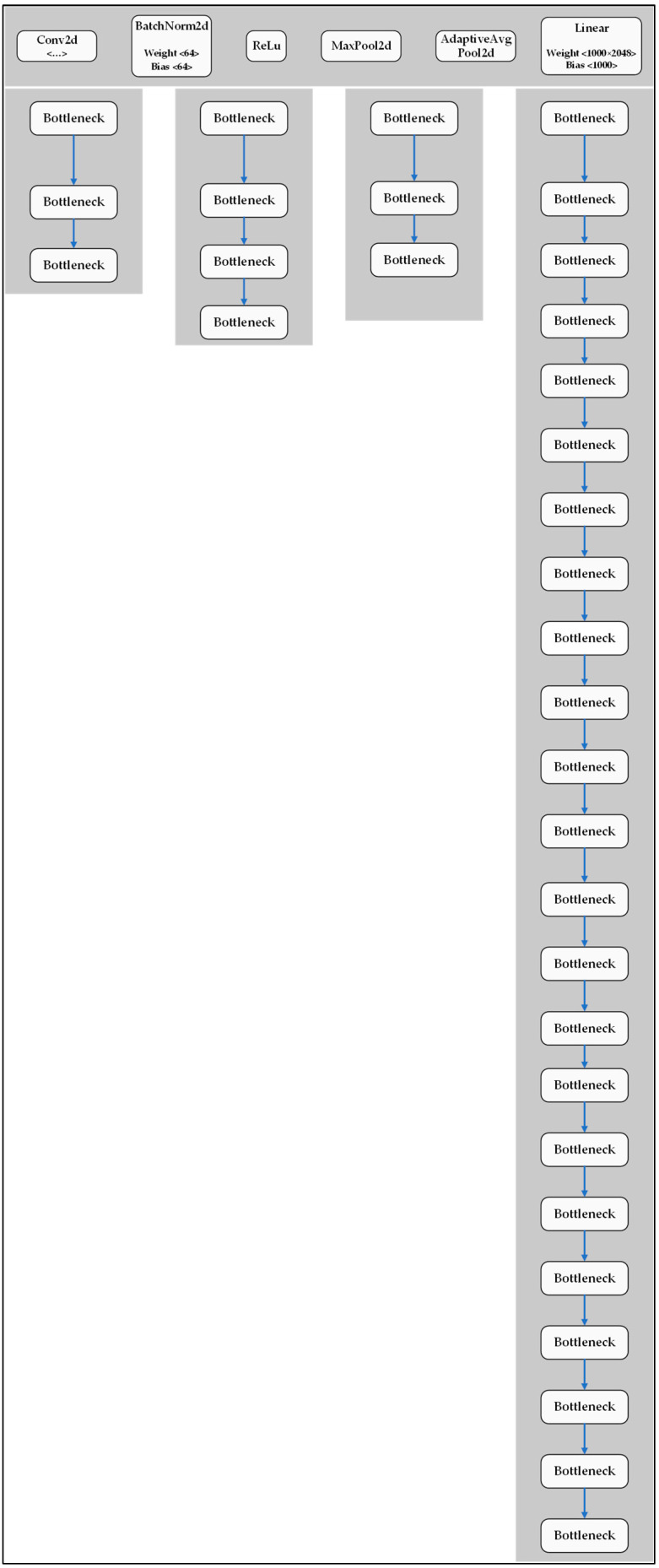
Structure of ResNet-101 model.

ResNet-101 has a similar structure to ResNet-50 but has fewer layers (101) and more parameters (45 million). As shown in Figure 12, ResNet-101 also uses bottleneck blocks to reduce the training time. The ResNet-101 model contains 287 layers, 42.5 million parameters, and 128.4 GFLOPs. In this study, the ResNet-101 model is run based on the YOLOv5 environment for the classification of tomatoes on the vine.

### 3.5. EfficientNet-B0

In recent studies, several groups have used EfficientNet to perform plant leaf disease classification and plant recognition [41,42]. There are eight versions of EfficientNet, ranging from B0 to B7, respectively. EfficientNet B0 achieved an accuracy of 77.1% on ImageNet, with 5.3 million parameters and 0.39B FLOPs, while ResNet-50 achieved an accuracy of 76%, with 26 million parameters and 4.1B FLOPs [43].

**Figure 13:**
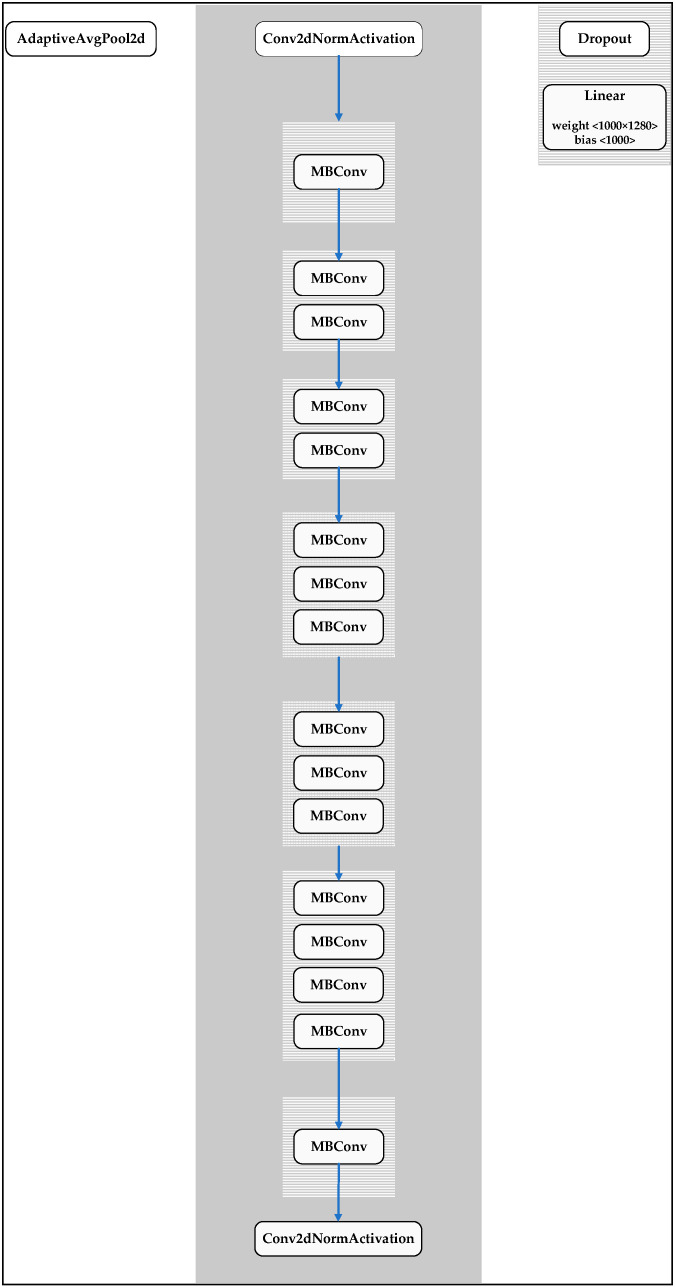
Structure of EfficientNet-B0.

As shown in Figure 13, the structure of EfficientNet-B0 consists of MBConv blocks, which are similar to the inverted residual blocks used in MobileNetv2 [44]. The blocks feature shortcut connections between the first and last sections of the block, and the input block is expanded by a 1 × 1 Conv layer to increase the number of channels or depth of the feature map. Conversely, the Depthwise Conv 3 × 3 and Pointwise Conv 1 × 1 layers are used to reduce the number of channels of the output block. The shortcut connections connect narrow layers, which have a small number of channels, while the wider layers are arranged between the shortcut connections. Notably, this structure reduces both the number of parameters and the number of operations. The EfficientNet-B0 model also uses the AdaptiveAvgPool2d layers to find important features of the data and reduce the training parameters. The dropout layer is used to reduce interdependent learning between neurons. The data regression layer is linear regression. Thus, even though EfficientNet-B0 has a large number of layers (337 layers), it has just 4 million parameters and 7.3 GFLOPs. In this study, the EfficientNet-B0 model is run based on the Yolov5 environment for the classification of tomatoes on the vine.

### 3.6. Confusion Matrix, Recall, Precision, Accuracy, F1 Score, and Rate

The testing performance of the various classification models was evaluated using the confusion matrix shown in Table 2. True positive (TP) is the number of predicted true positive or true positive results. True negative (TN) is the number of predicted true negative or true negative results. False positive (FP) is the number of predicted false positive or false positive results. False negative (FN) is number of predicted false negative or false negative results.

In the testing stage, the recall performance of the models was evaluated as the ratio of the number of samples correctly predicted as positive to the total number of samples predicted as positive, i.e.,

(1) $$\mathrm{Recall}=\frac{\mathrm{TP}}{\mathrm{TP}+\mathrm{FN}}$$

The precision was evaluated as the ratio of the number of samples correctly predicted as positive to the total number of positive predictions, i.e.,

(2) $$\mathrm{Precision}=\frac{\mathrm{TP}}{\mathrm{TP}+\mathrm{FP}}$$

The accuracy was defined as the ratio of the total number of correctly predicted samples to the total number of samples in the dataset, i.e.,

(3) $$\mathrm{Accuracy}=\frac{\mathrm{TP}+\mathrm{TM}}{\mathrm{TP}+\mathrm{TN}+\mathrm{FP}+\mathrm{FN}}$$

The F1 score was defined as the harmonic mean of the precision and recall, i.e.,

(4) $$F1=2\times \frac{\mathrm{Recall}\times \mathrm{Precision}}{\mathrm{Recall}+\mathrm{Precision}}$$

Rate is a measure factor in a confusion matrix. It has 4 types, including the true positive rate (TPR), true negative rate (TNR), false positive rate (FPR), and false negative ratee (FNR), i.e.,

(5) $$\mathrm{TPR}=\frac{\mathrm{TP}}{\sum \mathrm{Positive}}=\frac{\mathrm{TP}}{\mathrm{FN}+\mathrm{TP}}$$

(6) $$\mathrm{TNR}=\frac{\mathrm{TN}}{\sum \mathrm{Negative}}=\frac{\mathrm{TN}}{\mathrm{FP}+\mathrm{TN}}$$

(7) $$\mathrm{FPR}=\frac{\mathrm{FP}}{\sum \mathrm{Negative}}=\frac{\mathrm{FP}}{\mathrm{FP}+\mathrm{TN}}$$

(8) $$\mathrm{FNR}=\frac{\mathrm{FN}}{\sum \mathrm{Positive}}=\frac{\mathrm{FN}}{\mathrm{FN}+\mathrm{TP}}$$

### 3.7. Top 1 and Top 2 Accuracies

The effectiveness of the classification models in the training stage was further evaluated by means of the Top 1 and Top 2 accuracies. The Top 1 accuracy indicates the proportion of samples for which the category predicted by the model matches the true category. By contrast, the Top 2 accuracy considers the prediction result to be correct if either of the two most probable categories predicted by the model matches the true category.

### 3.8. Data Training

**Figure 14:**
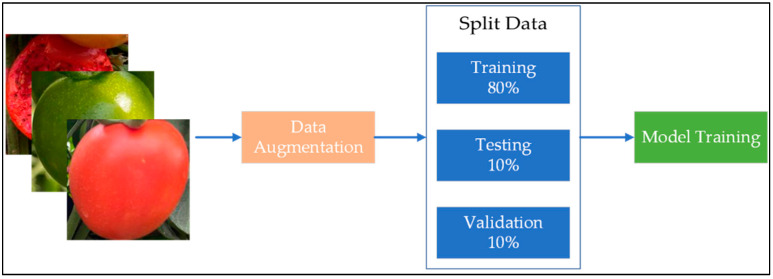
Flowchart showing division of the data augmentation dataset for model training.

The augmented dataset was split in the ratio of 80:10:10 for training, testing, and validation purposes, as shown in Figure 14. The computational properties of the training system and the training parameters are listed in Table 3 and Table 4, respectively.

## 4. Conclusions

This study proposed four deep learning frameworks (Yolov5m and Yolov5 with ResNet-50, ResNet-101, and EfficientNet-B0, respectively) for the classification of ripe, immature, and damaged tomatoes on the vine. The testing results showed that the ResNet-50, EfficientNet-B0, Yolov5m, and ResNet-101 models have overall accuracies of 98%, 98%, 97%, and 97%, respectively. Furthermore, all four models have a recall value of 100% for ripe tomato classification. The Yolov5m, ResNet-50, and ResNet-101 models also have a recall value of 1 for immature tomatoes. However, the recall value falls to 0.96 for the EfficientNet-B0 model. The recall values for damaged tomatoes vary in the range of 0.92 to 0.94. All four models achieve a precision of 0.98 for ripe tomatoes. The Yolov5m, ResNet-50, and ResNet-101 also have precisions of 1 for damaged tomatoes. However, the precision of the EfficientNet-B0 model falls to 0.95. The precision values of the four models for immature tomatoes vary in the range of 0.94–0.96. Finally, the four models all have F1 scores of 0.99 for ripe tomatoes. The F1 scores for immature and damaged tomatoes vary in the ranges of 0.96 to 0.98 and 0.93 to 0.97. The TNR and TPR have high values of 92–100% and 97–100%, respectively. While the FPR and FNR have low values of 0–3% and 0–8%, respectively. The model operates effectively. In general, the results confirm that all of the proposed models provide an accurate means of performing tomato fruit classification in automated fruit-harvesting applications.

## Figures and Tables

**Table 1:** Training model parameters and evaluation results of the four models.

Model	Layer	Parameters	GFLOPs	Top 1	Top 2	Time (hh:mm)
Yolov5m	212	11.7 M	30.9	0.997	1	00:52
YOLOv5-ResNet-50	151	23.5 M	67.5	0.993	1	00:58
YOLOv5-ResNet-101	287	42.5 M	128.4	0.997	1	01:13
YOLOv5-EfficientNet-B0	337	4.0 M	7.3	0.993	1	00:50

**Table 2:** Confusion matrix used for evaluating the classification accuracy.

	Predicts Label		
True Label		Positive	Negative
	Positive	TP (True Positive)	FN (False Negative)
	Negative	FP (False Positive)	TN (True Negative)

**Table 3:** The training system.

CPU	GPU	RAM
2 × Xeon Processors @2.3 Ghz, 46 MB Cache	Tesla P100 16 GB	16 GB

**Table 4:** Training parameter settings.

Parameter	Value
Optimization	Adam
Batch size	128
Learning rate	0.0001
Decay	5 × 10^−5^
Drop out	0.1
Epochs	100
Image size	224 × 224

## Data Availability

Data is available on request.

## References

[1] Linker R., Cohen O., Naor A. (2012). Determination of the number of green apples in RGB images recorded in orchards. Comput. Electron. Agric..

[2] Wei X., Jia K., Lan J., Li Y., Zeng Y., Wang C. (2014). Automatic method of fruit object extraction under complex agricultural background for vision system of fruit picking robot. Optik.

[3] Kelman E.E., Linker R. (2014). Vision-based localisation of mature apples in tree images using convexity. Biosyst. Eng..

[4] Payne A., Walsh K., Subedi P., Jarvis D. (2014). Estimating mango crop yield using image analysis using fruit at ‘stone hardening’stage and night time imaging. Comput. Electron. Agric..

[5] Qiang L., Jianrong C., Bin L., Lie D., Yajing Z. (2014). Identification of fruit and branch in natural scenes for citrus harvesting robot using machine vision and support vector machine. Int. J. Agric. Biol. Eng..

[6] Kurtulmus F., Lee W.S., Vardar A. (2014). Immature peach detection in colour images acquired in natural illumination conditions using statistical classifiers and neural network. Precis. Agric..

[7] Yamamoto K., Guo W., Yoshioka Y., Ninomiya S. (2014). On plant detection of intact tomato fruits using image analysis and machine learning methods. Sensors.

[8] Zhao Y., Gong L., Zhou B., Huang Y., Liu C. (2016). Detecting tomatoes in greenhouse scenes by combining AdaBoost classifier and colour analysis. Biosyst. Eng..

[9] Luo L., Tang Y., Zou X., Wang C., Zhang P., Feng W. (2016). Robust grape cluster detection in a vineyard by combining the AdaBoost framework and multiple color components. Sensors.

[10] Liu G., Mao S., Kim J.H. (2019). A mature-tomato detection algorithm using machine learning and color analysis. Sensors.

[11] Krizhevsky A., Sutskever I., Hinton G.E. (2012). Imagenet classification with deep convolutional neural networks. Adv. Neural Inf. Process. Syst..

[12] Szegedy C., Wei L., Yangqinh J., Pierre S., Scott R., Dragomir A., Dumitru E., Vincent V., Andrew R. Going deeper with convolutions. Proceedings of the IEEE Conference on Computer Vision and Pattern Recognition (CVPR).

[13] Simonyan K., Zisserman A. (2014). Very deep convolutional networks for large-scale image recognition. arXiv.

[14] Xie S., Girshick R., Dollár P., Tu Z., He K. Aggregated residual transformations for deep neural networks. Proceedings of the IEEE Conference on Computer Vision and Pattern Recognition.

[15] Zhang K., Wu Q., Liu A., Meng X. (2018). Can deep learning identify tomato leaf disease?. Adv. Multimed..

[16] Aversano L., Bernardi M.L., Cimitile M., Iammarino M., Rondinella S. Tomato diseases Classification Based on VGG and Transfer Learning. Proceedings of the 2020 IEEE International Workshop on Metrology for Agriculture and Forestry (MetroAgriFor).

[17] Karthik R., Hariharan M., Anand S., Mathikshara P., Johnson A., Menaka R. (2020). Attention embedded residual CNN for disease detection in tomato leaves. Appl. Soft Comput..

[18] Girshick R., Donahue J., Darrell T., Malik J. Rich feature hierarchies for accurate object detection and semantic segmentation. Proceedings of the IEEE Conference on Computer Vision and Pattern Recognition.

[19] Ren S., He K., Girshick R., Sun J. (2016). Faster R-CNN: Towards real-time object detection with region proposal networks. IEEE Trans. Pattern Anal. Mach. Intell..

[20] Dai J., Li Y., He K., Sun J. (2016). R-FCN: Object detection via region-based fully convolutional networks. Adv. Neural Inf. Process. Syst..

[21] Liu W., Anguelov D., Erhan D., Szegedy C., Reed S., Fu C.Y., Berg A.C. Ssd: Single shot multibox detector. Proceedings of the European Conference on Computer Vision.

[22] Redmon J., Divvala S., Girshick R., Farhadi A. You only look once: Unified, real-time object detection. Proceedings of the IEEE Conference on Computer Vision and Pattern Recognition.

[23] Hu C., Liu X., Pan Z., Li P. (2019). Automatic detection of single ripe tomato on plant combining faster R-CNN and intuitionistic fuzzy set. IEEE Access.

[24] Fuentes A., Yoon S., Kim S.C., Park D.S. (2017). A robust deep-learning-based detector for real-time tomato plant diseases and pests recognition. Sensors.

[25] Mirhaji H., Soleymani M., Asakereh A., Mehdizadeh S.A. (2021). Fruit detection and load estimation of an orange orchard using the YOLO models through simple approaches in different imaging and illumination conditions. Comput. Electron. Agric..

[26] Chen J., Wang Z., Wu J., Hu Q., Zhao C., Tan C., Teng L., Luo T. (2021). An improved Yolov3 based on dual path network for cherry tomatoes detection. J. Food Process Eng..

[27] Elsayed E., Amany S. (2016). Evaluation of nutritional value and antioxidant activity of tomato peel extracts. Arab. J. Chem..

[28] Lin H.T. (2017). Cherry tomato ‘TSS ASVEG No.22’.

[29] Wang P., Niu T., He D. (2021). Tomato Young Fruits Detection Method under Near Color Background Based on Improved Faster R-CNN with Attention Mechanism. Agriculture.

[30] Zu L., Zhao Y., Liu J., Su F., Zhang Y., Liu P. (2021). Detection and Segmentation of Mature Green Tomatoes Based on Mask R-CNN with Automatic Image Acquisition Approach. Sensors.

[31] Lawal M.O. (2021). Tomato detection based on modified YOLOv3 framework. Sci. Rep..

[32] Liu G., Nouaze J.C., Touko Mbouembe P.L., Kim J.H. (2020). YOLO-Tomato: A Robust Algorithm for Tomato Detection Based on YOLOv3. Sensors.

[33] Xiao J.R., Chung P.C., Wu H.Y., Phan Q.H., Yeh J.L.A., Hou M.T.K. (2020). Detection of strawberry diseases using a convolutional neural network. Plants.

[34] Wang C.Y., Bochkovskiy A., Liao H.Y.M. Scaled-yolov4: Scaling cross stage partial network. Proceedings of the IEEE/cvf Conference on Computer Vision and Pattern Recognition.

[35] Zhu X., Lyu S., Wang X., Zhao Q. TPH-YOLOv5: Improved YOLOv5 based on transformer prediction head for object detection on drone-captured scenarios. Proceedings of the IEEE/CVF International Conference on Computer Vision.

[36] Chen Z., Wu R., Lin Y., Li C., Chen S., Yuan Z., Chen S., Zou X. (2022). Plant Disease Recognition Model Based on Improved YOLOv5. Agronomy.

[37] Kamilaris A., Prenafeta-Boldú F.X. (2018). A review of the use of convolutional neural networks in agriculture. J. Agric. Sci..

[38] Mukti I., Biswas D. Transfer Learning Based Plant Diseases Detection Using ResNet50. Proceedings of the 2019 4th International Conference on Electrical Information and Communication Technology (EICT).

[39] Reddy A.S.B., Juliet D.S. Transfer learning with ResNet-50 for malaria cell-image classification. Proceedings of the 2019 International Conference on Communication and Signal Processing (ICCSP).

[40] He K., Zhang X., Ren S., Sun J. Deep residual learning for image recognition. Proceedings of the IEEE Conference on Computer Vision and Pattern Recognition.

[41] Ümit A., Murat U., Kemal A., Emine U. (2021). Plant leaf disease classification using EfficientNet deep learning model. Ecol. Inform..

[42] Arun Y., Viknesh G.S. Leaf Classification for Plant Recognition Using EfficientNet Architecture. Proceedings of the 2022 IEEE Fourth International Conference on Advances in Electronics, Computers and Communications (ICAECC).

[43] Tan M., Le Q. Efficientnet: Rethinking model scaling for convolutional neural networks. Proceedings of the 36th International Conference on Machine Learning.

[44] Sandler M., Howard A., Zhu M., Zhmoginov A., Chen L.C. Mobilenetv2: Inverted residuals and linear bottlenecks. Proceedings of the IEEE Conference on Computer Vision and Pattern Recognition.

